# The mechanism of (+) taxifolin’s protective antioxidant effect for •OH-treated bone marrow-derived mesenchymal stem cells

**DOI:** 10.1186/s11658-017-0066-9

**Published:** 2017-12-27

**Authors:** Xican Li, Hong Xie, Qian Jiang, Gang Wei, Lishan Lin, Changying Li, Xingmei Ou, Lichan Yang, Yulu Xie, Zhen Fu, Yamei Liu, Dongfeng Chen

**Affiliations:** 1 School of Chinese Herbal Medicine, Guangzhou University of Chinese Medicine, Waihuang East Road No. 232, Guangzhou Higher Education Mega Center, Guangzhou, 510006 China; 2 Innovative Research & Development Laboratory of TCM, Guangzhou University of Chinese Medicine, Guangzhou, 510006 China; 3 School of Basic Medical Science, Guangzhou University of Chinese Medicine, Guangzhou, 510006 China; 4 The Research Center of Integrative Medicine, Guangzhou University of Chinese Medicine, Guangzhou, 510006 China

**Keywords:** (+) Taxifolin; bmMSCs, •OH damage, Antioxidant mechanism, Electron transfer, Fe^2+^ binding

## Abstract

The natural dihydroflavonol (+) taxifolin was investigated for its protective effect on Fenton reagent-treated bone marrow-derived mesenchymal stem cells (bmMSCs). Various antioxidant assays were used to determine the possible mechanism. These included •OH-scavenging, 2-phenyl-4, 4, 5, 5-tetramethylimidazoline-1-oxyl-3-oxide radical-scavenging (PTIO•-scavenging), 1, 1-diphenyl-2-picryl-hydrazl radical-scavenging (DPPH•-scavenging), 2, 2′-azino-bis (3-ethylbenzo-thiazoline-6-sulfonic acid) radical-scavenging (ABTS^+^•-scavenging), Fe^3+^-reducing, and Cu^2+^-reducing assays. The Fe^2+^-binding reaction was also investigated using UV-Vis spectra. The results revealed that cell viability was fully restored, even increasing to 142.9 ± 9.3% after treatment with (+) taxifolin. In the antioxidant assays, (+) taxifolin was observed to efficiently scavenge •OH, DPPH• and ABTS^+^• radicals, and to increase the relative Cu^2+^- and Fe^3+^-reducing levels. In the PTIO•-scavenging assay, its IC_50_ values varied with pH. In the Fe^2+^-binding reaction, (+) taxifolin was found to yield a green solution with two UV-Vis absorbance peaks: λ_max_ = 433 nm (ε =5.2 × 10^2^ L mol^−1^ cm ^−1^) and λ_max_ = 721 nm (ε = 5.1 × 10^2^ L mol^−1^ cm ^−1^). These results indicate that (+) taxifolin can act as an effective •OH-scavenger, protecting bmMSCs from •OH-induced damage. Its •OH-scavenging action consists of direct and indirect antioxidant effects. Direct antioxidation occurs via multiple pathways, including ET, PCET or HAT. Indirect antioxidation involves binding to Fe^2+^.

**Electronic supplementary material:**

The online version of this article (10.1186/s11658-017-0066-9) contains supplementary material, which is available to authorized users.

## Background

Antioxidant supplementation has been suggested as a means to reduce the DNA damage and relieve oxidative stress during the expansion and proliferation of bone marrow-derived mesenchymal stem cells (bmMSCs) [1]. This oxidative stress is a result of the imbalance between ROS production and diminished endogenous antioxidant protection. Accumulative ROS (especially •OH with a half-life of 10^−9^ s) not only have the potential to damage all types of biomolecules (such as DNA, proteins, lipids and carbohydrates), but can also inhibit MSC immunomodulation, thus increasing senescence and reducing ex vivo expansion, which is critical for clinical application off the cells [2]. Effective antioxidants that could protect MSCs from oxidative stress are a desirable focus of research.

From the perspective of free radical biology, plants also encounter serious oxidative stress from strong UV-Vis light, atmospheric ROS, temperature changes, and the processes of oxygen consumption for photosynthesis. Notably, some plants, such as pine, have what could be considered a strong vital force and a long history of survival. They have successfully resisted oxidation from complicated ecological environments and may serve as a library of efficient phenolic antioxidants [3].

**Fig. 1:**
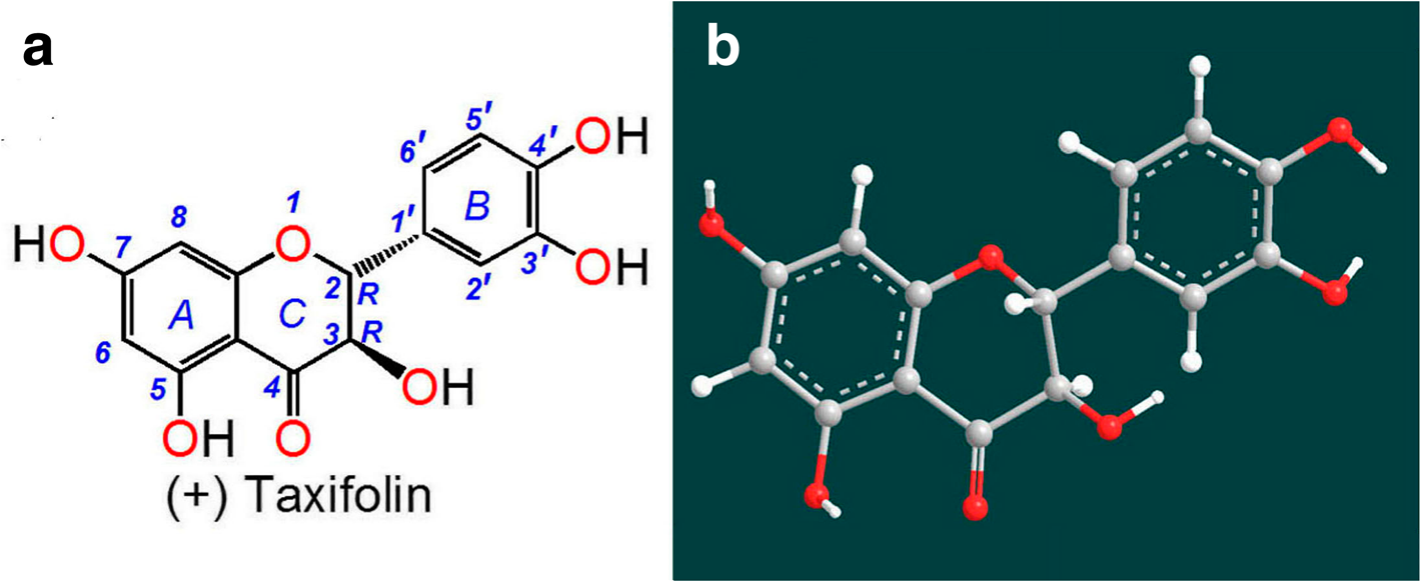
Structure (**a**) and preferential conformation-based ball-and-stick model (**b**) of (+) taxifolin

Pine grows on the Sharon Plain in Israel and in mountains and highlands around the world. Notable species and varieties are *Pinus pinaster* (French maritime pine) [4], *Pseudotsug amenziesii* [5], *Pinus massoniana* Lamb [6], *Pinus sylvestris var.* mongovica Litvin [7] and *Larix olgensis* Henry var. Koreana Nakai [8]. Pine has survived for approximately 1.9 hundred million years, suggesting that it possesses strong defenses, probably including a strong antioxidant defense with numerous antioxidant components. In fact, extract from the bark of French maritime pine has been developed as an antioxidant supplement known commercially as Pycnogenol, which has a bioactive component named (+) taxifolin (*2R,3R*–dihydroquercetin, Fig. 1) [4, 9].

As shown in Fig. 1a, (+) taxifolin is actually a dihydroflavonol that exists in the aforementioned pine types. It was reported to inhibit free radical formation at key stages of apoptosis in cellular mitochondria [10] and to correct cerebral ischemia-reperfusion injury [11]. Recently, (+) taxifolin was found to exhibit anticancer and neuroprotective effects [12–14].

This indicates that (+) taxifolin has potential as an antioxidant for protecting MSCs against oxidative stress damage. However, no study has reported on the protective effects of (+) taxifolin towards •OH-treated bmMSCs.

Here, we applied the methyl thiazolyl tetrazolium (MTT) assay to assess the protective effects of (+) taxifolin on •OH-treated bmMSCs. We then explored the possible mechanisms for this effect.

## Methods

### Chemicals and animals

The chemicals (+) taxifolin (CAS number: 480–18-2, 98%), dihydromyricetin (CAS number: 27,200–12-0, 98%), and 4’-*O*-methyltaxifolin (CAS number: 70,411–27-7, 98%) were obtained from Chengdu Biopurify Phytochemicals Ltd. Catechol (CAS number: 120–80-9, 99.5%) and DNA sodium salt (fish sperm) were purchased from Aladdin Chemistry Co. The 1, 1-diphenyl-2-picryl-hydrazyl radical (DPPH•), (±)-6-hydroxyl-2, 5, 7, 8-tetramethylchromane-2-carboxylic acid (trolox), 2, 9-dimethyl-1, 10-phenanthroline (neocuproine), 3-(2-pyridyl)-5, 6-bis (4-phenylsulfonicacid)-1, 2, 4-triazine (ferrozine), 2, 4, 6-tripyridyltriazine (TPTZ), 2-phenyl-4, 4, 5, 5-tetramethylimidazoline-1-oxyl-3-oxide radical (PTIO•), and methyl thiazolyl tetrazolium (MTT) were purchased from Sigma-Aldrich Shanghai Trading Co. The CCK-8 (BB-4221-2) kits were from BestBio Inc. (NH_4_)_2_ABTS [2, 2′-azino-bis (3-ethylbenzo-thiazoline-6-sulfonic acid diammonium salt)] was obtained from Amresco Chemical Co. Dulbecco’s modified Eagle’s medium (DMEM), fetal bovine serum (FBS) and trypsin were purchased from Gibco. All other reagents were of analytical grade.

Sprague-Dawley (SD) rats (4 weeks old) were obtained from the Animal Center of Guangzhou University of Chinese Medicine. The protocol was performed under the supervision of the Institutional Animal Ethics Committee at the Guangzhou University of Chinese Medicine.

### MTT assay to assess the protective effect against •OH-induced damage

The bmMSCs were cultured according to our previous report [15] with slight modifications. In brief, bone marrow was obtained from the femur and tibia of the rats. Marrow samples were diluted with low-glucose DMEM containing 10% FBS. MSCs were prepared by gradient centrifugation at 900×***g*** for 30 min on 1.073 g/ml Percoll. The prepared cells were detached by treatment with 0.25% trypsin and passaged in culture flasks at 1 × 10^4^/cm^2^. At passage 3, bmMSCs were evaluated for cell homogeneity using CD44 detection via flow cytometry. These cells were used for the subsequent experiments.

The protective effect of (+) taxifolin against •OH-induced bmMSC damage was investigated based on the method described in [16, 17] with slight modifications. Briefly, bmMSCs were seeded at 5000 cells per well into 96-well plates. After adherence for 24 h, bmMSCs were divided into control, model and sample [(+) taxifolin] groups.

In the control group, bmMSCs were incubated for 24 h in DMEM. In the model and sample groups, bmMSCs were incubated in the presence of FeCl_2_ (100 μM) followed by H_2_O_2_ (50 μM). After incubation for 20 min, the mixture of FeCl_2_ and H_2_O_2_ was removed. The bmMSCs in the model group were incubated for 24 h in DMEM, while bmMSCs in the sample group were incubated for 24 h in DMEM with the indicated (+) taxifolin concentrations.

After incubation, 20 μl MTT (5 mg/ml) was added, and the culture was incubated for an additional 3 h. The culture medium was discarded and replaced with 150 μl DMSO. Absorbance was measured at 490 nm on a Bio-Kinetics reader (PE-1420; Bio-Kinetics Corporation). Culture medium containing serum was used for the control group and each sample test was repeated in five independent wells.

### Hydroxyl-scavenging assay based on DNA

The hydroxyl-scavenging effect of (+) taxifolin was estimated using a method developed by our laboratory [18]. Briefly, methanol sample solutions (1.2 mg/ml, 20–100 μl) were separately aliquoted into mini tubes. After completely evaporating the methanol solvent in each tube to dryness, the sample residue was treated with 300 μl of phosphate buffer (0.2 M, pH 7.4), followed by 50 μl of DNA sodium (10 mg/ml), 75 μl of H_2_O_2_ (33.6 mM), 50 μl of FeCl_3_ (3.2 mM), 100 μl of Na_2_EDTA (0.5 mM) and 75 μl of ascorbic acid (12 mM). After incubation at 50 °C for 20 min, 250 μl of trichloroacetic acid (10%, *w*/*v*) was added to the tube. After heating the mixture at 105 °C for 15 min with 150 μl of 2-thiobarbituric acid (TBA, 5% in 1.25% NaOH aqueous solution), the absorbance was measured using a Unico Spectrophotometer UV 2100 against the buffer (blank). The protective effect is expressed as follows:

$$ Protective effect\%=\frac{A_0\hbox{-} A}{A_0}\times 100\%, $$

where A_0_ indicates the absorbance of the blank and A indicates the absorbance of the sample (+) taxifolin.

### PTIO•-scavenging assay

The PTIO•-scavenging assay was conducted based on our method [19]. In brief, 80 μl of an aqueous PTIO• solution (0.1 mM) was mixed with 20 μl of phosphate buffer at pH 5.0, 6.0, 7.0, 7.4, 8.0 and 9.0 containing 1 mg/ml of sample at the indicated concentrations. The mixture was maintained at 37 °C for 30 min, and the absorbance was measured at 560 nm on a microplate reader (Multiskan FC, Thermo Scientific). The PTIO• inhibition percentage was calculated as follows:

$$ Scavenging\%=\frac{A_0\hbox{-} A}{A_0}\times 100\%, $$

where A_0_ indicates the absorbance of the blank and A indicates the absorbance of the sample, (+) taxifolin.

### DPPH•-scavenging assay and ABTS^+^•-scavenging assay

DPPH• radical-scavenging activity was determined as previously described [20]. Briefly, 1 ml of DPPH• solution (0.1 M) was mixed with the indicated concentrations of sample (0.15 mg/ml, 14–70 μl) dissolved in methanol. The mixture was maintained at room temperature for 30 min, and the absorbance was measured at 519 nm on a Unico Spectrophotometer 2100.

ABTS^+^•-scavenging activity was evaluated according to a previously described method [21]. ABTS^+^• was produced by mixing 0.2 ml of ABTS diammonium salt (7.4 mM) with 0.35 ml of potassium persulfate (2.6 mM). The mixture was maintained in the dark at room temperature for 12 h to allow completion of radical generation and then diluted with 95% ethanol. To determine the scavenging activity, the test sample (x = 15–75 μl, 0.03 mg/ml) was added to (200- x) μl of 95% ethanol followed by 800 μl of ABTS^+^• reagent, and the absorbance was measured at 734 nm on a Unico Spectrophotometer 2100 6 min after the initial mixing using 95% ethanol as the blank.

The percentage of DPPH•-scavenging (or ABTS^+^•-scavenging) activity was calculated based on the formula given in the **PTIO•-scavenging assay** section.

### Cu^2+^-reducing assay

The reducing power capacity of cupric ions (Cu^2+^) was measured according to a previously described method [22] with a slight modification. Briefly, 125 μl of CuSO_4_ aqueous solution (10 mM), 125 μl of neocuproine ethanolic solution (7.5 mM) and 750 μl of CH_3_COONH_4_ buffer solution (0.1 M, pH 7.5) were added to test tubes with different volumes of sample (0.15 mg/ml, 15–75 μl). The total volume was adjusted to 1 ml with buffer and mixed vigorously. The absorbance against a buffer blank was measured at 450 nm after 30 min. An increase in the absorbance of the reaction mixture indicates an increase in reduction capability. The relative reducing power of the sample relative to the maximum absorbance was calculated using the following formula:

$$ Relative reducing effect\%=\frac{A\hbox{-} {A}_{min}}{A_{max}\hbox{-} {A}_{min}}\times 100\%, $$

where A_min_ is the absorbance of the control without sample, A is the absorbance of the reaction mixture with sample, and A_max_ is the maximum absorbance of the reaction mixture with sample.

### Ferric-reducing antioxidant power (FRAP) assay

The FRAP assay was adapted from Benzie and Strain [23]. Briefly, FRAP reagent was freshly prepared by mixing 10 mM TPTZ, 20 mM FeCl_3_ and 0.25 M acetate buffer at 1:1:10 (pH 3.6). The test sample (x = 20–100 μl, 0.5 mg/ml) was added to (100- x) μl of 95% ethanol followed by 400 μl of FRAP reagent. The absorbance was measured at 593 nm after a 30-min incubation at ambient temperature using distilled water as the blank. The relative reducing power was calculated using the formula given in the **Cu** ^**2+**^ **-reducing assay** section*.*

### UV-vis spectra and color reaction of Fe^2+^-binding

The (+) taxifolin–Fe^2+^ complex was evaluated using UV-Vis spectroscopy. For these experiments, 300 μl of a methanolic solution of (+) taxifolin and 100 μl of an aqueous solution of FeCl_2_•4H_2_O were added to 600 μl of an aqueous mixture of distilled water and methanol (1:1). The solution was then mixed vigorously and continuously scanned using a UV-Vis spectrophotometer (Unico 2600A) from 200 to 900 nm after 0, 10, 20, 30, and 60 min.

The above experiment was repeated using 4’-O-methyltaxifolin.

### Statistical analysis

Each experiment was performed in triplicate and data were recorded as the means ± SD (standard deviation). Dose response curves were plotted using Origin 6.0 software (OriginLab). IC_50_ was defined as the final concentration of 50% radical inhibition (relative reducing power or binding effect). Statistical comparisons were made using one-way ANOVA to detect significant differences using SPSS 13.0 (SPSS Inc.) for Windows. *p* < 0.05 was considered statistically significant.

## Results and discussion

**Fig. 2:**
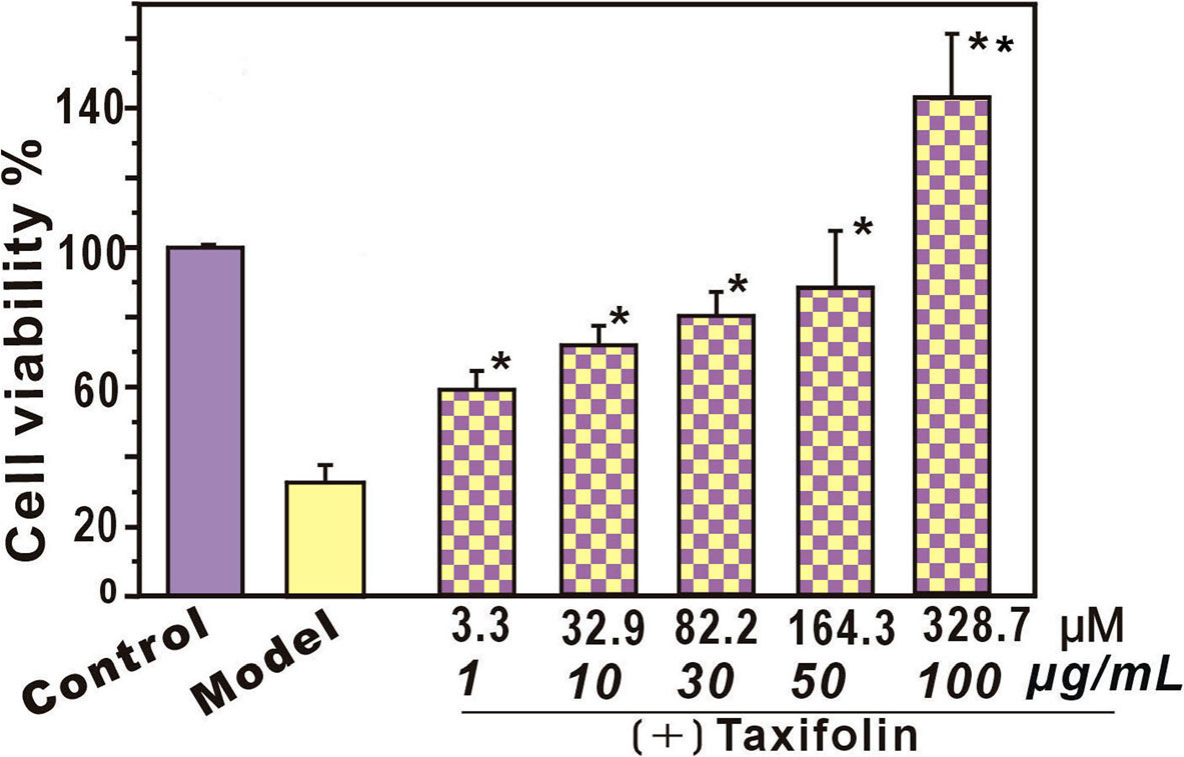
Protective effect of (+) taxifolin towards •OH-treated bmMSCs determined using the MTT assay. The •OH radical was generated via the addition of Fenton reagent (FeCl_2_•4H_2_O) followed by H_2_O_2_. Each value is expressed as the mean ± SD, *n* = 3; *p < 0.05 vs. model (MSCs damaged by •OH radical). **p < 0.05 vs. control

As shown in Fig. 2, in the model group, the bmMSCs damaged by •OH presented only 33.1 ± 4.4% viability. However, following treatment with (+) taxifolin (3.3–164.3 μM), cell viability was restored or even increased. This result suggests that (+) taxifolin effectively protects bmMSCs from •OH-mediated damage. This is consistent with the recent report that (+) taxifolin could reduce cholesterol oxidation product-induced neuronal apoptosis [24]. At higher concentrations (>50 μg/ml, 164.3 μM), (+) taxifolin could even further promote the viability of bmMSCs, reaching 142.9 ± 9.3% viability.

To test the possible toxicity to MSCs, the effect of (+) taxifolin towards normal MSCs was measured using the CCK-8 assay (an updated version of the MTT assay). The results indicated that (+) taxifolin (3.3–328.7 μM) had no effect on proliferation and no toxic effect on normal MSCs without •OH-treatment (Additional file 1: Figure S1). These results align with the previous findings that (+) taxifolin could be an apparent exception that could efficiently inhibit the Fenton reaction and superoxide radical formation [25, 26] while being completely nonphototoxic, unlike its analogue quercetin [13, 27]. These results are inconsistent with another previous study that showed taxifolin was toxic to oocytes at higher concentration (50 μg/ml, 164.3 μM) [28].

It is assumed that when (+) taxifolin was mixed with Fenton reagents, some reaction products may be generated to bring about the beneficial (especially protective) effect. In fact, a similar situation is observed with salvianolic acid B, which can increase cell viability to 175.1% [29]. In the case of the salvianolic acid B molecule, some characteristic chemical structures, such as catechol or lactone moieties [29], have been suggested to be partly responsible for the protective effect. The moiety of fused rings (A/B) is also hypothesized to play a role in the process. Some antioxidants comprising 8-hydroxyquinol have been demonstrated to induce MSC proliferation [30, 31]. However, the detailed mechanisms should be investigated further.

Such protective effects from •OH damage have been reported to be related to •OH scavenging [32]. In this study, (+) taxifolin was found to exhibit •OH-scavenging ability in a dose-dependent manner (Additional file 1: Figure S2). The IC_50 Trolox_/IC_50 (+) taxifolin_ value (1.67; Table 1) suggests that (+) taxifolin is a better •OH scavenger than trolox, which is a standard antioxidant.

**Table 1:** The IC_50_ values of (+) taxifolin and trolox in various assays (μM)

Assays	(+) taxifolin	Trolox	Ratio value	
•OH-scavenging	259.2 ± 4.4 ^a^	411.4 ± 17.0 ^b^	1.59	Average 1.67
PTIO•-scavenging*	663.9 ± 34.4 ^a^	736.8 ± 29.9 ^b^	1.11	
DPPH•-scavenging	16.0 ± 0.2 ^a^	18.5 ± 0.4 ^b^	1.16	
ABTS^+^•-scavenging	4.6 ± 0.2 ^a^	11.4 ± 0.2 ^b^	2.48	
Cu^2+^-reducing	22.4 ± 0.5 ^a^	40.4 ± 1.9^b^	1.80	
FRAP	33.7 ± 1.0 ^a^	62.8 ± 1.0 ^b^	1.86	

The IC_50_ value was defined as the final concentration of 50% radical inhibition (relative reducing power). It was calculated by linear regression analysis, and expressed as the mean ± SD (n = 3). The linear regression was analyzed using Origin 6.0. Mean values with different superscripts (a or b) in the same row are significantly different (p < 0.05). *The assay was conducted at pH 7.4. The ratio value is defined as IC_50 Trolox_/IC_50 (+) taxifolin_. The dose–response curves are shown in Additional file 1: Figures S2-S7

•OH scavenging comprises two pathways: direct and indirect. The direct antioxidant pathway directly scavenges the •OH free radical that has been generated via the Fenton reaction. However, •OH is a very transient species so it is impossible to verify whether •OH is directly scavenged. Therefore, we used a stable oxygen-centered radical, PTIO•, for the investigation. As seen in Additional file 1: Figure S3A, (+) taxifolin scavenged the PTIO• radical at various pH values in a dose-dependent manner.

Correspondingly, the IC_50_ values varied with various pH values: 2.6 ± 0.5, 1.6 ± 0.2, 0.7 ± 0.03, 0.6 ± 0.04, 0.5 ± 0.04 and 0.4 ± 0.02 mM respectively for pH 5.0, 6.0, 7.0, 7.4, 8.0 and 9.0 (Additional file 1: Table S1). This indicates the involvement of the direct antioxidant pathway in •OH scavenging by (+) taxifolin. When the IC_50_ values were plotted against pH values, a first-order decay curve was observed (Additional file 1: Figure S3B), suggesting that a high level of H^+^ (low pH value) considerably suppressed the PTIO•-scavenging ability of (+) taxifolin. Thus, the radical-scavenging ability of (+) taxifolin is hypothesized to be involved in H^+^ transfer, consistent with the cyclic voltammetry-based evidence [33].

It has been documented that at a pH ≤ 5.0, PTIO• can be scavenged via electron transfer (ET) [33]. Our assay suggests that (+) taxifolin may also scavenge PTIO• at pH 5.0, indicating the involvement of ET in its antioxidant action. This is further supported by its ABTS^+^•-scavenging, Cu^2+^-reducing and Fe^3+^-reducing (i.e., FRAP) abilities (Additional file 1: Figures S4-S6). ABTS^+^•-scavenging is considered to be an ET-based pathway [34]. The ABTS^+^•-scavenging ability of (+) taxifolin indicates the involvement of ET in the antioxidant process. Furthermore, (+) taxifolin increased the relative Cu^2+^-reducing and FRAP-reducing abilities in a concentration-dependent manner. The FRAP (at pH 3.6) and Cu^2+^-reducing activities have been demonstrated to be an ET reaction [35]. It should be noted that the Fe^3+^-reducing potential of flavonoids may also reduce Fe^3+^ into Fe^2+^ to cause pro-antioxidation [36]. It remains unknown whether the pro-antioxidation is linked to (+) taxifolin cytotoxicity to oocytes at higher concentration [28].

In this study, (+) taxifolin efficiently scavenged the DPPH· radical (Additional file 1: Figure S7). DPPH· scavenging is regarded as a hydrogen atom transfer-based multi-pathway [32]. Successful DPPH· scavenging by (+) taxifolin indicated that hydrogen atom transfer may occur in its direct antioxidative process. Moreover, it was recently reported that these direct antioxidative pathways are not exclusive but are rather competitive based on various reaction conditions [34].

**Fig. 3:**
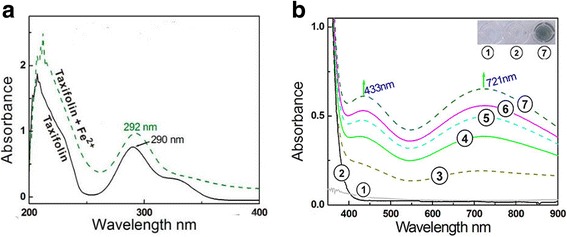
The UV-vis spectra of (+) taxifolin and its Fe^2+^-complex. **a** Comparison of UV spectra of 0.05 mmol/l (+) taxifolin and 0.05 mmol/l (+) taxifolin *plus* 2.5 mmol/l Fe^2+^. **b** Vis spectra of 1.0 mmol/l (+) taxifolin and Vis spectra of the reaction mixtures of 1.0 mmol/l (+) taxifolin with 50.0 mmol/l Fe^2+^ for 0, 10, 20, 30, 60 min (① 50.0 mmol/l Fe^2+^; ② 1.0 mmol/l (+) taxifolin; ③ reaction mixture for 0 min; ④ reaction mixture for 10 min; ⑤ reaction mixture for 20 min; ⑥ reaction mixture for 30 min; ⑦ reaction mixture for 60 min. The inset in Fig. 3B is the appearance of solutions

Because Fe^2+^ can catalyze the Fenton reaction, where H_2_O_2_ yields •OH radicals, an attenuation of Fe^2+^ levels via a binding reaction is considered an indirect antioxidant mechanism to scavenge •OH radicals [37]. In the indirect antioxidant assay, (+) taxifolin bound to Fe^2+^ to yield a green solution and two Vis absorbance peaks: λ_max_ = 433 nm (ε =5.2 × 10^2^ L mol^−1^ cm ^−1^) and λ_max_ = 721 nm (ε = 5.1 × 10^2^ L mol^−1^ cm ^−1^). In the UV spectra, Fe^2+^ binding enhanced the peak strength around 290 nm (Fig. 3). These results strongly indicate a binding reaction between Fe^2+^ and (+) taxifolin and that Fe^2+^ binding may act as one indirect pathway in the antioxidative process of (+) taxifolin.

**Fig. 4:**
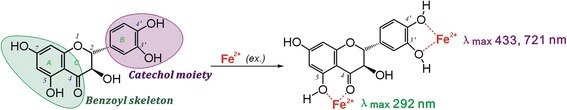
Proposed reaction of (+) taxifolin binding to Fe^2+^ (including UV-Vis spectra assignments)

As reported previously [36], adjacent keto or hydroxyl groups are potential targets of Fe^2+^ binding, while isolated keto-group (or hydroxyl-group) cannot bind iron. Nevertheless, the 3, 4-hydroxyl-keto moiety cannot give a planar conformation (Fig. 1b), and can barely form the planar five-membered Fe^2+^-complex. As a dihydroflavonol, (+) taxifolin contains only two Fe^2+^-binding sites: the 3′, 4′-catechol moiety and the 4, 5-hydroxyl-keto moiety (Fig. 4) [38].

Despite several reports on the metal-binding of flavonoids [38–40] and descriptions of Na^+^ interacting with flavonoids [41], studies focusing on UV-Vis spectral analyses (especially peak assignment) are lacking. To confirm the assignment of the UV-Vis peaks in Fig. 3, we investigated the Fe^2+^-binding of catechol and dihydromyricetin (reference compounds), because in (+) taxifolin and dihydromyricetin, the possible π–π conjugation is blocked by a single 2, 3 carbon–carbon bond, and the *B* ring and *A/C* fused rings are independent of each other. Thus, the whole (+) taxifolin molecule can be divided into two spectroscopic systems: the benzoyl skeleton and the catechol moiety (Additional file 1: Figure S8). Catechol contains a similar chemical structure to the *B* ring of (+) taxifolin, while dihydromyricetin bears a similar chemical structure to the *A/C* fused rings (benzoyl skeleton) of (+) taxifolin.

Catechol–Fe^2+^ gave two similar absorbance peaks (at approximately λ_max_ 433 and 721 nm) in the Vis spectra to those of (+) taxifolin-Fe^2+^ and yielded a green solution (Additional file 1: Figure S9). By contrast, the dihydromyricetin molecule bearing a pyrogallol moiety in the *B* ring presented a strong absorbance peak at λ_max_ 589 nm [42], and 4’-*O*-methyltaxifolin without catechol moiety only gave a UV absorbance peak at λ_max_ 289 nm (Additional file 1: Figure S10). Thus, it can be deduced that the peaks in the Vis spectra of the (+) taxifolin–Fe^2+^ complex are from the Fe^2+^-binding reaction with catechol in the *B* ring.

With respect to the UV spectra, an enhanced strength of the UV peaks was observed in the Fe^2+^-binding reaction with (+) taxifolin (Fig. 3a), similar to the dihydromyricetin–Fe^2+^ complex and 4’-*O*-methyltaxifolin–Fe^2+^ complex (Additional file 1: Figures S8 & S11). Dihydromyricetin and 4’-*O*-methyltaxifolin share a similar benzoyl skeleton with (+) taxifolin. Thus, the enhancement of peak around 290 nm can be attributed to the Fe^2+^-binding reaction of the 4-hydroxyl-5-keto moiety. This assumption is further supported by the different colors between the (+) taxifolin–Fe^2+^ complex and the 4’-*O*-methyltaxifolin–Fe^2+^ complex.

## Conclusion

As an effective •OH-scavenger, (+) taxifolin can protect bmMSCs from •OH-induced damage. Its •OH-scavenging action consists of direct and indirect antioxidant effects. The direct antioxidation occurs via multiple pathways, including ET, PCET and HAT. The indirect antioxidation involved Fe^2+^ binding. Upon binding to Fe^2+^, the 3′,4′-catechol moiety in the *B* ring gives rise to two peaks (λ_max_ 433 nm and 721 nm), and the 4-hydroxyl-5-keto of the benzoyl skeleton causes an enhanced peak intensity around 290 nm.

## Additional file

Additional file 1: Figure S1. The CCK-8 assay for normal bmMSCs exposed to (+) taxifolin. **Figure S2.** Dose–response curves for (+) taxifolin •OH-scavenging assay based on DNA. **Figure S3.** Dose–response curves for (+) taxifolin in PTIO• radical-scavenging assay and its IC_50_ values at various pH values. **Figure S4.** Dose–response curves for (+) taxifolin in the ABTS^+^• radical-scavenging assay. **Figure S5.** Dose–response curves for (+) taxifolin in the Cu^2+^-reducing assay. **Figure S6.** Dose–response curves for (+) taxifolin in the FRAP assay. **Figure S7.** Dose–response curves for (+) taxifolin in the DPPH•-radical-scavenging assay. **Figure S8.** The UV-visible spectra for the 4’-O-methyltaxifolin–Fe^2+^ complex. **Figure S9.** The UV absorption bands of flavonoid. **Figure S10.** The UV-Vis spectra and solution colors for (+) taxifolin–Fe^2+^ and catechol–Fe^2+^. **Figure S11.** The UV-Vis spectra for (+) taxifolin–Fe^2+^ and dihydromyricetin–Fe^2+^. **Table S1.** The IC_50_ values listed in different units. (DOCX 789 kb)

## Abbreviations

ABTS: 2, 2′-azino-bis (3-ethylbenzo-thiazoline-6-sulfonic acid)
bmMSCs: bone marrow-derived mesenchymal stem cells
CCK-8: Cell counting kit-8
DMEM: Dulbecco’s modified Eagle’s medium
ET: Electron transfer
FBS: Fetal bovine serum
Ferrozine: 3-(2-pyridyl)-5,6-bis (4-phenylsulfonicacid)-1,2,4-triazine
FRAP: Ferric ion-reducing antioxidant power
HAT: Hydrogen atom transfer
MTT: Methyl thiazolyl tetrazolium
Neocuproine: 2, 9-dimethyl-1, 10-phenanthroline
PCET: Proton-coupled electron transfer
PTIO•: 2-phenyl-4,4,5, 5-tetramethylimidazoline-1-oxyl-3-oxide
ROS: Reactive oxygen species
TPTZ: 2,4,6-tripyridyl triazine
Trolox: (±)-6-hydroxyl-2,5,7,8-tetramethlychromane-2-carboxylic acid
