# Supplementary material for: The mechanism of (+) taxifolin’s protective antioxidant effect for •OH-treated bone marrow-derived mesenchymal stem cells
Source: Cell Mol Biol Lett. 2017 Dec 27;22:31. doi: 10.1186/s11658-017-0066-9 (PMC5745628; doi:10.1186/s11658-017-0066-9)
Supplement: Additional file 1: Figure S1. — The CCK-8 assay for normal bmMSCs exposed to (+) taxifolin. Figure S2. Dose–response curves for (+) taxifolin •OH-scavenging assay based on DNA. Figure S3. Dose–response curves for (+) taxifolin in PTIO• radical-scavenging assay and its IC50 values at various pH values. Figure S4. Dose–response curves for (+) taxifolin in the ABTS+• radical-scavenging assay. Figure S5. Dose–response curves for (+) taxifolin in the Cu2+-reducing assay. Figure S6. Dose–response curves for (+) taxifolin in the FRAP assay. Figure S7. Dose–response curves for (+) taxifolin in the DPPH•-radical-scavenging assay. Figure S8. The UV-visible spectra for the 4’-O-methyltaxifolin–Fe2+ complex. Figure S9. The UV absorption bands of flavonoid. Figure S10. The UV-Vis spectra and solution colors for (+) taxifolin–Fe2+ and catechol–Fe2+. Figure S11. The UV-Vis spectra for (+) taxifolin–Fe2+ and dihydromyricetin–Fe2+. Table S1. The IC50 values listed in different units. (DOCX 789 kb) [file 11658_2017_66_MOESM1_ESM.docx]

**Preventive effect and mechanism of (+) taxifolin as antioxidant towards •OH-treated bone marrow-derived mesenchymal stem cells**

Xican Li ^1, 2,*†^, Hong Xie ^1, 2†^, Qian Jiang ^1^, Gang Wei ^1, 2^, Lishan Lin ^1^, Changying Li ^1^, Xingmei Ou ^1^, Lichan Yang ^1^, Yulu Xie ^1, 2^, Zhen Fu^3, 4^, Yamei Liu^3, 4^, Dongfeng Chen ^3, 4, *^

^1^ School of Chinese Herbal Medicine, ^2^ Innovative Research & Development Laboratory of TCM, ^3^ School of Basic Medical Science, ^4^ The Research Center of Integrative Medicine, Guangzhou University of Chinese Medicine, Guangzhou, China, 510006.


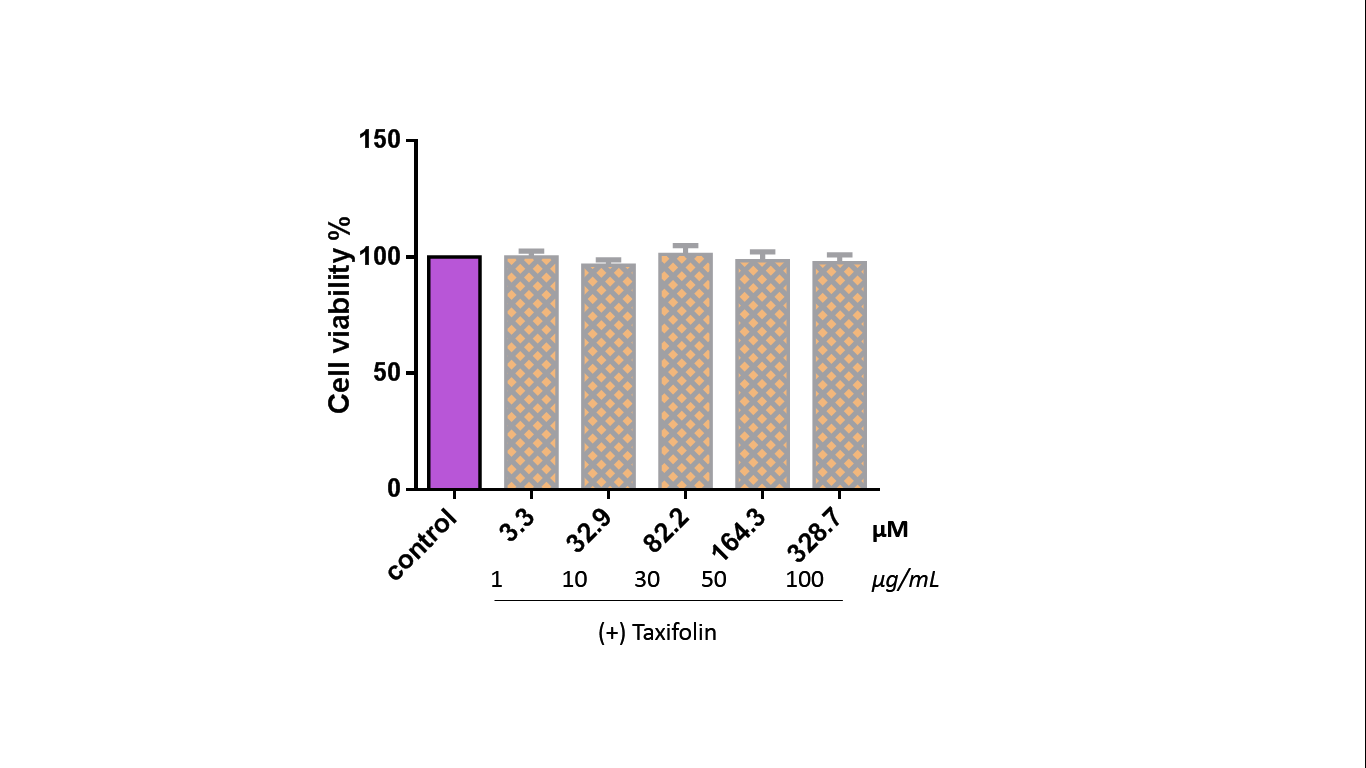


**Fig. S1.** The effect of (+) taxifolin towards normal bmMSCs without treatment of •OH radical (CCK-8 assay) Each value is expressed as mean±SD, n=5

(bmMSCs were seeded at 5000 cells per well into 96-well plates. After adherence for 24 hours, bmMSCs were divided into control, and sample [(+) taxifolin] groups. In the control group, bmMSCs were incubated for 24 hours in DMEM; while bmMSCs in the sample group were incubated for 24 hours in DMEM with the indicated (+) taxifolin concentrations. After incubation, 10 μL CCK-8(BB-4221-2, BestBio) was added, and the culture was incubated for an additional 2 hours. Absorbance was measured at 450 nm on a Bio-Kinetics reader (PE-1420; Bio-Kinetics Corporation, Sioux Center, IA, USA). Culture medium containing serum was used for the control group and each sample test was repeated in three independent wells.)


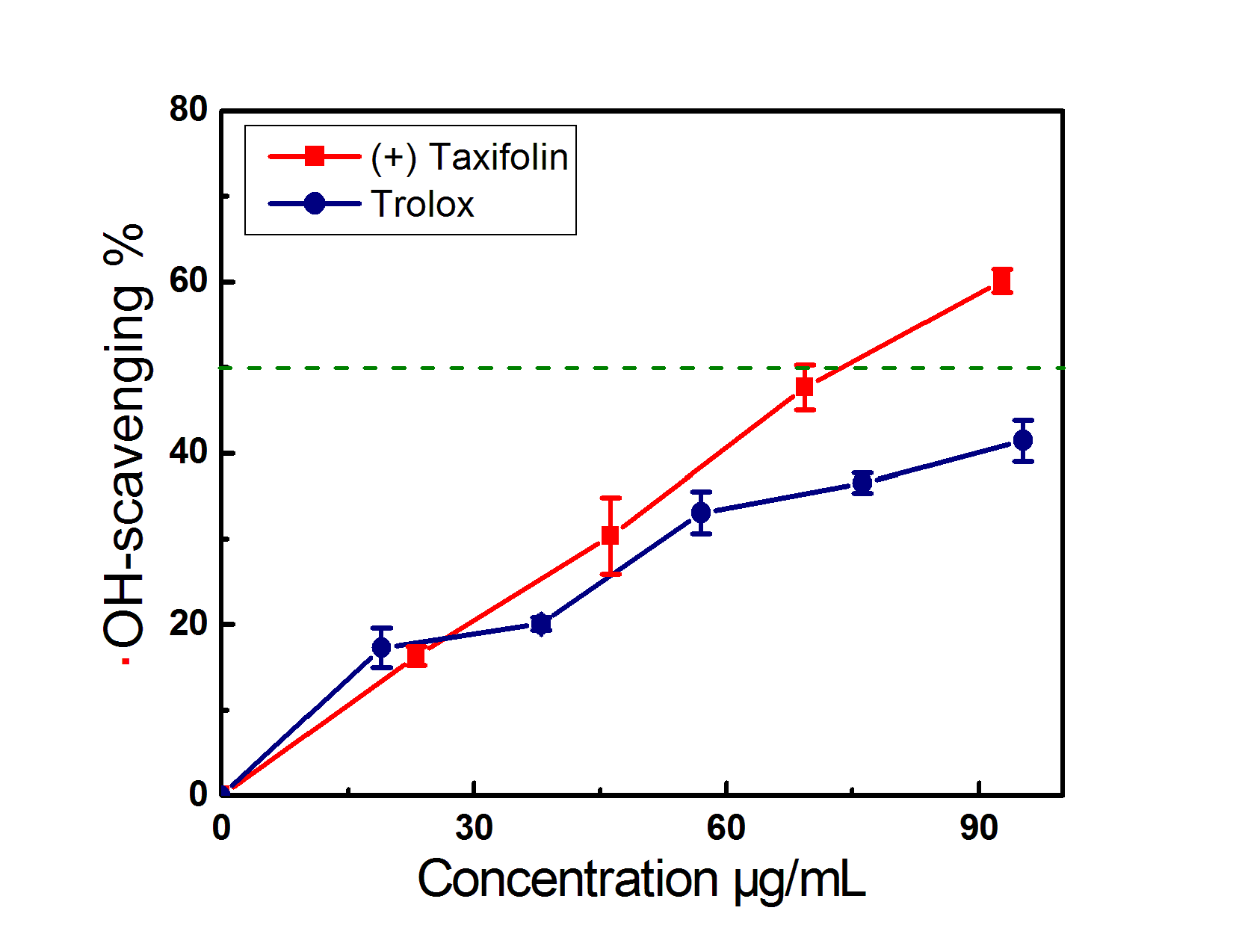


**Fig. S2.** Dose response curves of (+)taxifolin and Trolox in Hydroxyl-scavenging assay based on DNA. Each value is expressed as the mean±SD, *n*=3.


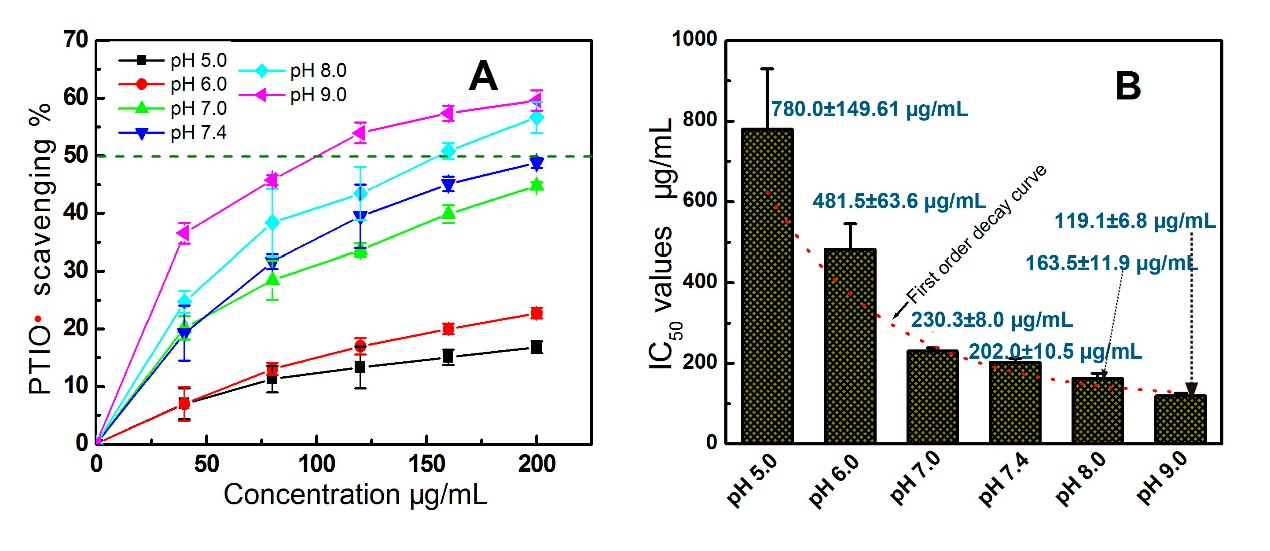


**Fig. S3. (A)**Dose response curves of (+) Taxifolin in PTIO•-radical-scavenging assay; **(B)**The IC_50_ values of (+) taxifolin at various pH values. Each value is expressed as the mean±SD, *n*=3.

T**able S1** The IC_50_ values listed in different units

| pH values | pH 5.0 | pH 6.0 | pH 7.0 | pH 7.4 | pH 8.0 | pH 9.0 |
| --- | --- | --- | --- | --- | --- | --- |
| IC_50_ (µg/mL) | 780.0±149.6 | 481.5±63.6 | 230.3±8.0 | 202.0±10.5 | 163.5±11.9 | 119.1±6.8 |
| IC_50_ (mM) | 2.6±0.5 | 1.6±0.2 | 0.7±0.03 | 0.6±0.04 | 0.5±0.04 | 0.4±0.02 |

***
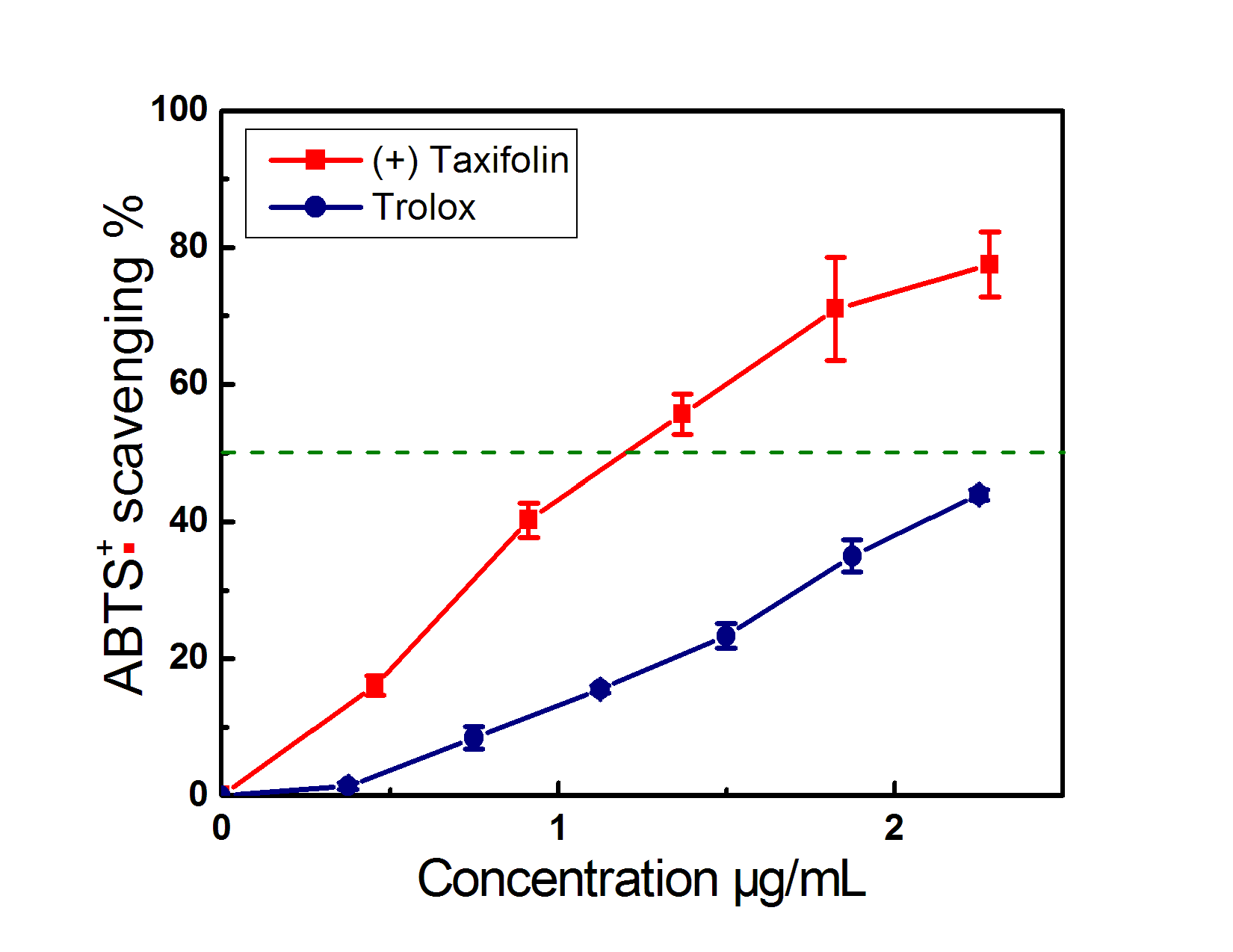
***

**Fig. S4.** Dose response curves of (+) Taxifolin and Trolox in ABTS•^+^ radical-scavenging assay. Each value is expressed as the mean±SD, *n*=3.


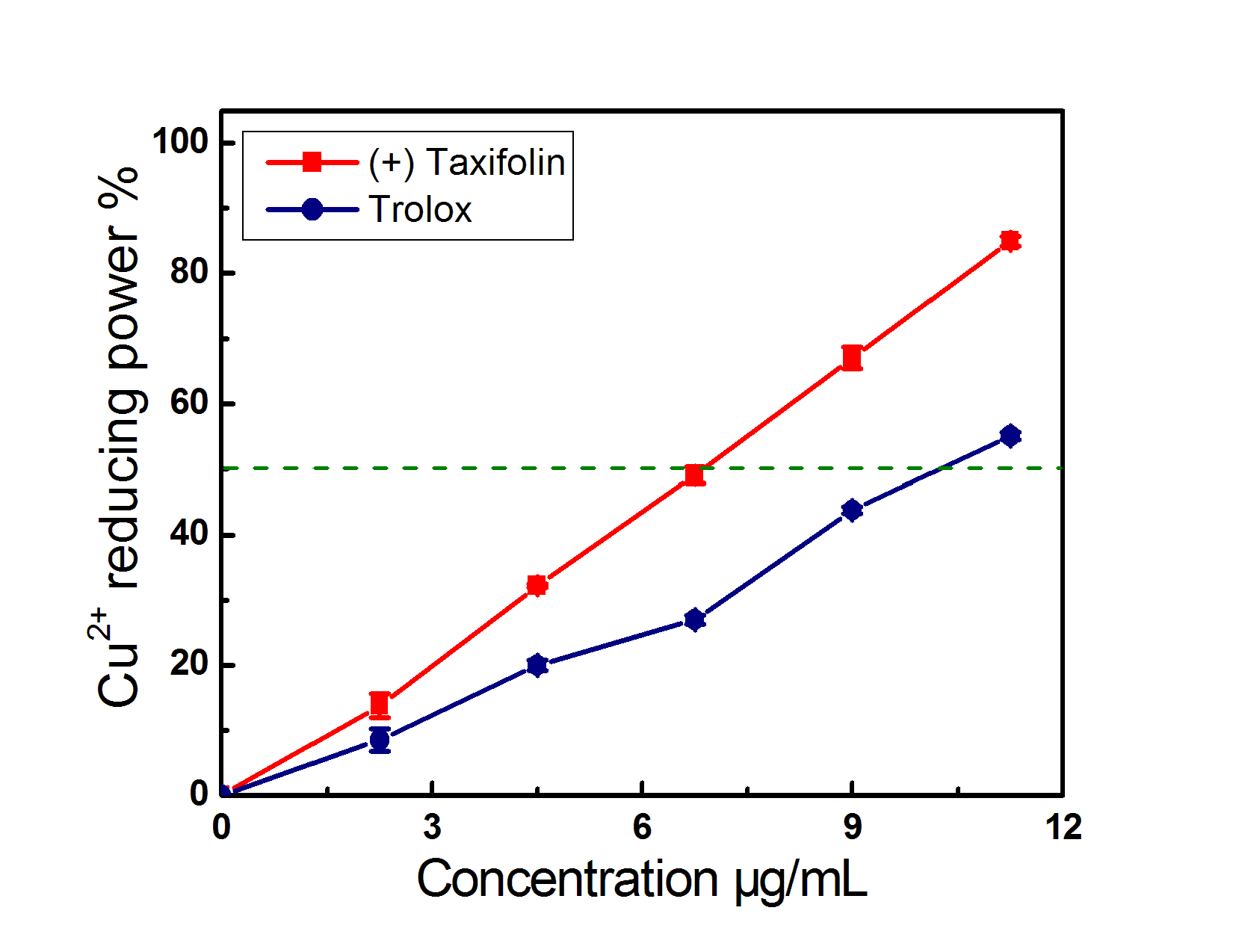


**Fig. S5.** Dose response curves of (+) taxifolin and Trolox in the Cu^2+^-reducing assay. Each value is expressed as the mean±SD, *n*=3.

*
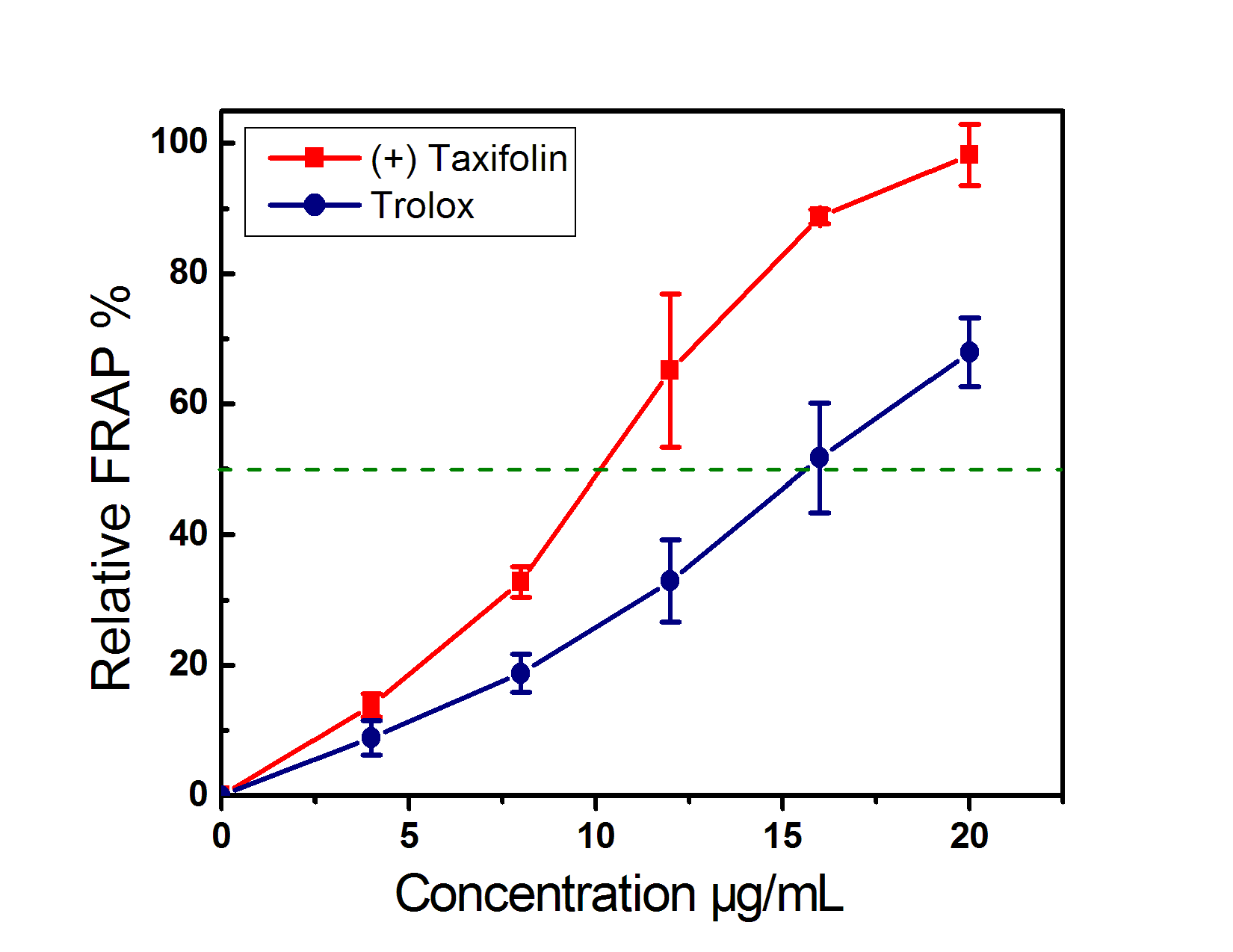
*

**Fig. S6.** Dose response curves of (+) Taxifolin and Trolox in the FRAP assay. Each value is expressed as the mean±SD, *n*=3.

***
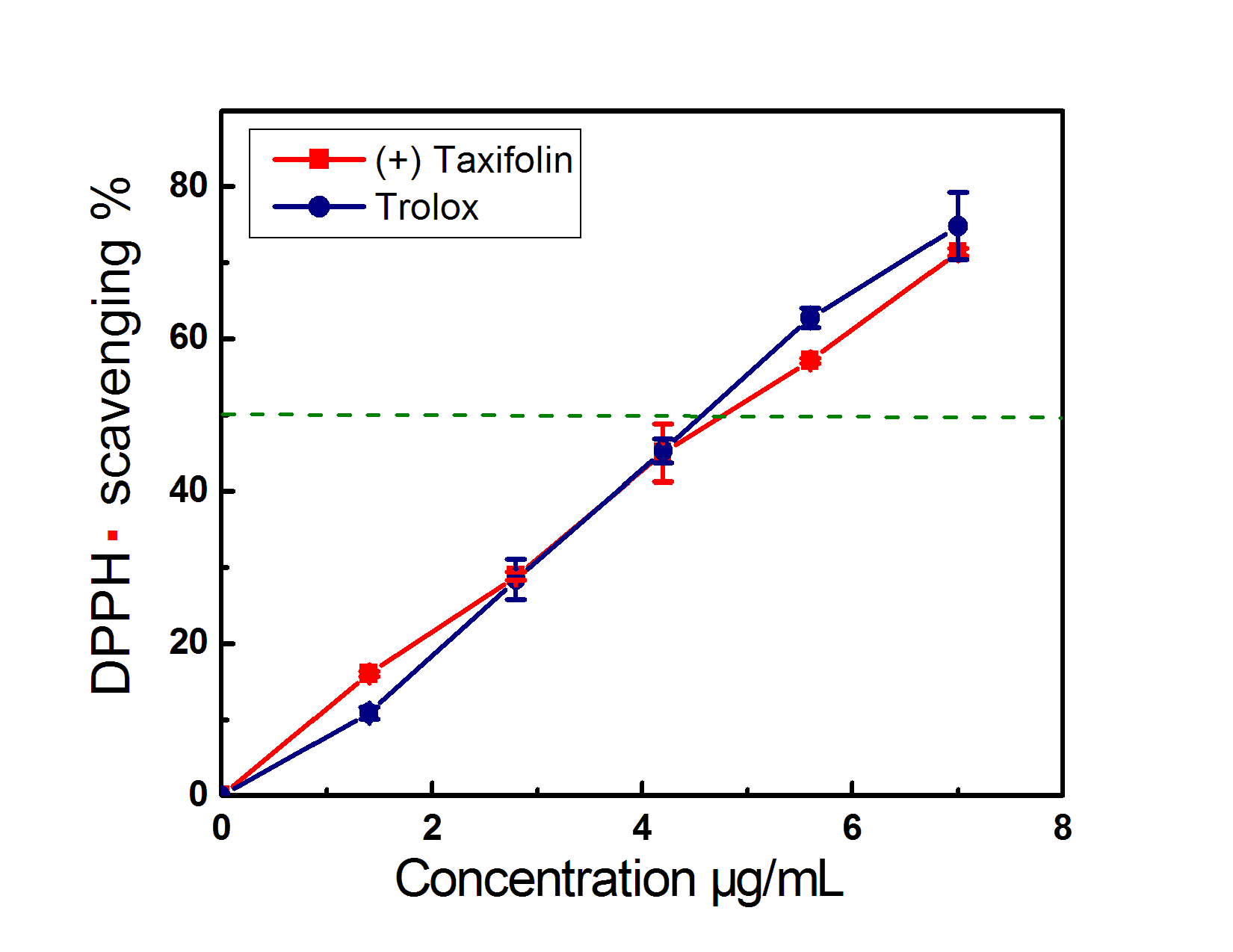
***

**Fig. S7.** Dose response curves of (+) Taxifolin and Trolox in DPPH•-radical-scavenging assay. Each value is expressed as the mean±SD, *n*=3.


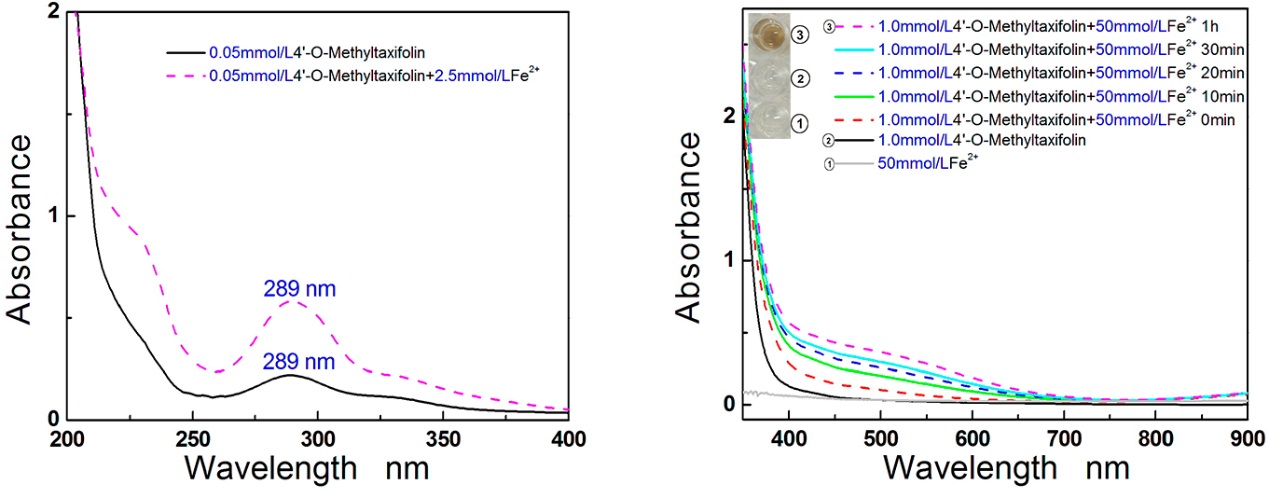


**Fig. S8.** The UV-visible spectra of 4'-O-methyltaxifolin-Fe^2+^ complex


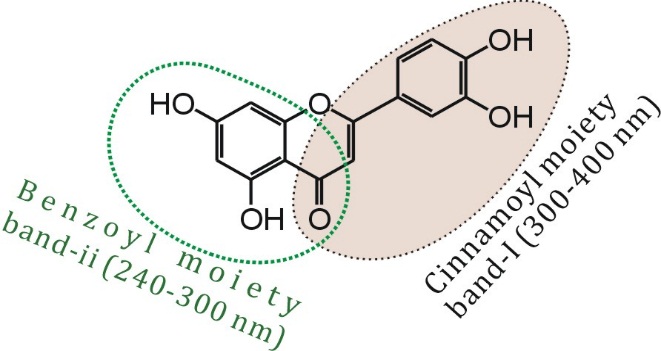


**Fig. S9.** The UV absorption bands of flavonoid


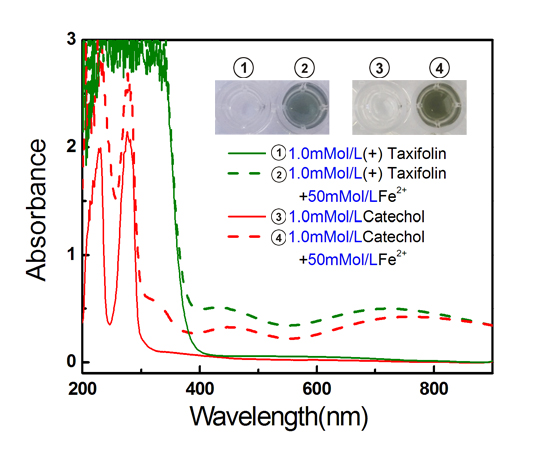


**Fig. S10.** The UV-Vis-spectra and solution colors of (+)taxifolin-Fe^2+^ and catechol-Fe^2+^

^

^

**Fig. S11.** UV-Vis-spectra of (+) taxifolin-Fe^2+^ and dihydromyricetin-Fe^2+^
